# Identification and Characterization of a Trillin Rhamnosyltransferase From *Dioscorea zingiberensis*

**DOI:** 10.3389/fpls.2021.713036

**Published:** 2021-08-06

**Authors:** Jia Li, Isidore Mosongo, Han Li, Yalun Wu, Changfu Li, Shihui Yang, Yansheng Zhang

**Affiliations:** ^1^State Key Laboratory of Biocatalysis and Enzyme Engineering, Environmental Microbial Technology Center of Hubei Province, School of Life Sciences, Hubei University, Wuhan, China; ^2^CAS Key Laboratory of Plant Germplasm Enhancement and Specialty Agriculture, Wuhan Botanical Garden, Chinese Academy of Sciences, Wuhan, China

**Keywords:** *Dioscorea zingiberensis*, steroidal saponin, dioscin biosynthesis, prosapogenin A of dioscin, UDP-rhamnosyltransferase, trillin rhamnosyltransferase (DzGT1)

## Abstract

Dioscorea zingiberensis accumulates abundant steroidal saponins, such as dioscin, which is the principal bioactive ingredient displaying a wide range of pharmacological activities. Diosgenin is the aglycone of dioscin, and recently, genes encoding cytochrome P450 enzymes in the late steps of diosgenin biosynthesis have been isolated. Diosgenin was successfully synthesized in the cholesterol-producing yeasts. From diosgenin to dioscin, one glucose and two rhamnose groups need to be added. Although genes encoding UDP-glucosyltransferases converting diosgenin to trillin were isolated, genes encoding UDP-rhamnosyltransferases involved in dioscin biosynthesis remain unknown. In this study, we isolated the cDNA encoding the trillin rhamnosyltransferase (designated DzGT1) from *D. zingiberensis*. Heterologous expression of DzGT1 in *Escherichia coli* cells showed that the gene product exhibits an enzyme activity that glycosylates the trillin to form prosapogenin A of dioscin (PSA). The transcript level of *DzGT1* is in accord with PSA accumulation in different organs of *D. zingiberensis*. Integration of the biochemical, metabolic, and transcriptional data supported the function of DzGT1 in dioscin biosynthesis. The identification and characterization of DzGT1 will help understand the metabolism of steroidal saponins in *D. zingiberensis* and provide candidate UDP-rhamnosyltransferase for efficient production of PSA, dioscin, and relevant steroidal saponins in microbial hosts.

## Introduction

*Dioscorea zingiberensis*, belonging to the family of Dioscoreaceae, is a traditional medicinal plant mainly distributed in Shaanxi, Hubei, Sichuan, Hunan, and Gansu provinces in China. Traditionally, its rhizomes were used to treat bee stings, lung heat, cough, bruises, and soft tissue injuries (Zhang et al., 2018). Currently, it is widely used for the treatment of cardiovascular diseases (Qin et al., 2009). Steroidal saponins extracted from the rhizomes of *D. zingiberensis* are the major bioactive constituents possessing a wide range of biological activities, including anti-thrombotic activity (Li et al., 2010), neuroprotective effect (Zhang et al., 2014), cardioprotective effect (Tang et al., 2015), and anthelmintic activity (Wang et al., 2010). Among these steroidal saponins, dioscin is the principal active component. In recent years, dioscin has received significant attentions due to its pharmacological activities, such as anti-tumor, anti-inflammatory, anti-hyperuricemia, antifungal, antiviral, cardioprotective, nephroprotective, and hepatoprotective properties (Tao et al., 2018; Yang et al., 2019).

Despite the medicinal importance of dioscin, its whole biosynthetic pathway has not been elucidated yet. Diosgenin is the aglycone of dioscin, and previous studies showed that cholesterol might be the precursor for diosgenin biosynthesis (Joly et al., 1969; Stohs et al., 1969). Later, the biosynthetic pathway of cholesterol was discovered in plants (Sonawane et al., 2016). Recently, genes encoding P450 enzymes in the late steps of diosgenin biosynthesis have been isolated from *Paris polyphylla*, *Trigonella foenum–graecum*, and *D. zingiberensis* (Christ et al., 2019; Cheng et al., 2021). By integrating the steroid C-16, 22-dihydroxylase, and C-26 hydroxylase genes, diosgenin was successfully synthesized in the cholesterol-producing yeasts (Christ et al., 2019; Cheng et al., 2021). These studies provided the basis for the heterologous biosynthesis of dioscin. As long as the glycosyltranferases synthesizing dioscin are found, heterologous biosynthesis of dioscin is most likely realized.

From diosgenin to dioscin, one glucose and two rhamnose groups need to be added at the C-3 position of diosgenin by UDP-glucosyltransferase and UDP-rhamnosyltransferase. Although genes encoding UDP-glucosyltransferase genes that convert diosgenin to its C-3 glycosylated product trillin were isolated from *D. zingiberensis* (Ye et al., 2017; Li et al., 2018), genes encoding UDP-rhamnosyltransferase involved in the biosynthesis of dioscin and other steroidal saponins have not been isolated and characterized yet. Only some UDP-rhamnosyltransferases in the biosynthetic pathways of steroidal glycoalkaloids and triterpene saponins whose structure is similar to steroidal saponins have been studied. For instance, GmSGT3 transferring a rhamnosyl group from UDP-rhamnose to soyasaponin III to form the triterpene saponin soyasaponin I in *Glycine* max (Shibuya et al., 2010), and StSGT3 as the β-solanine/β-chaconine rhamnosyltransferase, playing a role in the formation of the potato steroidal glycoalkaloids (McCue et al., 2007). Therefore, the UDP-rhamnosyltransferase gene resource of steroidal saponins is limited. It is crucial to carry out research work to isolate and characterize genes encoding UDP-rhamnosyltransferases to expand the gene resource of UDP-rhamnosyltransferases for the biosynthesis of steroidal saponins.

**FIGURE 1:**
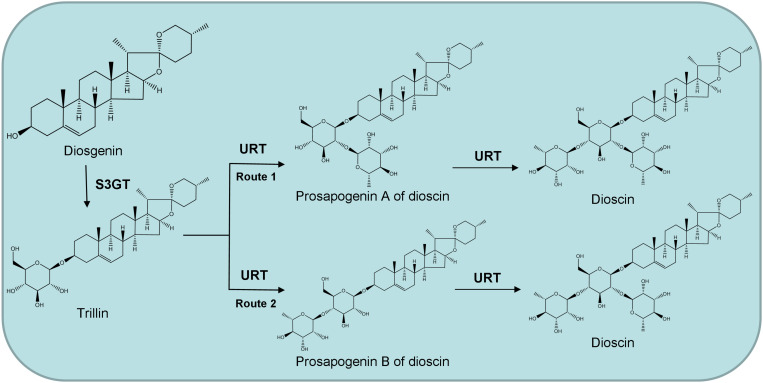
Proposed two possible pathways for dioscin biosynthesis in *Dioscorea zingiberensis*. S3GT, sterol 3-*O*-glucosyltransferase; URT, UDP-rhamnosyltransferase.

The UDP-rhamnosyltransferase genes involved in dioscin biosynthesis are unknown, and the order of addition of the two rhamnose groups in dioscin production is unclear, too. We speculate that dioscin is synthesized in two different pathways, which are illustrated in Figure 1. Either the prosapogenin A of dioscin (PSA) or the prosapogenin B of dioscin (PSB) may be the direct precursor of dioscin. Therefore, further study is needed to identify and characterize the rhamnosyltransferases involved in dioscin biosynthesis and confirm the biosynthetic pathway of the sugar chain. In this study, we utilized the transcriptomic datasets from the leaves and rhizomes of *D. zingiberensis* to isolate the UDP-rhamnosyltransferase genes involved in dioscin biosynthesis and four candidate UGTs were cloned and characterized. One of the UGTs, DzGT1, was found to be a UDP-rhamnosyltransferase, glycosylating trillin to form the product PSA. The identification of DzGT1 provides the basis for heterogenous biosynthesis of PSA, dioscin, and relevant steroidal saponins in other plants and microbial hosts.

## Materials and Methods

### Plant Materials and Chemical

Wild-type *D. zingiberensis* plants were collected from Zhuxi County, Hubei province of China. The healthy rhizomes were planted at 25°C with 14/10 h of a light/dark cycle. The chemical standards, UDP-glucose and UDP-rhamnose, were purchased from Shanghai Source Leaf Biological Technology Company (China). High-performance liquid chromatography (HPLC) grade acetonitrile and methanol (Thermo Fisher Scientific, United States) were used for HPLC and liquid chromatography-mass spectrometry (LC-MS). The restriction endonucleases and vector pMD18-T were from Takara Company (China). ClonExpress^®^ II One Step Cloning Kit was purchased from Vazyme Biotech Co., Ltd. (China).

### Determination of UGT Candidates

The transcriptome derived from the leaves and rhizomes of *D. zingiberensis* was constructed previously (Li et al., 2018). The biosynthesis of dioscin requires the addition of glucose and rhamnose groups onto the C-3 hydroxyl group, catalyzed by UDP-glycosyltransferases (UGTs). BLAST searching against several public databases was done, and 40 unigenes were annotated as UGTs, which are involved in the metabolic process with a sequence length above 1,500 bps, and 34 full-length genes were obtained from the 40 unigenes. Out of the 34 genes, two genes, c61522_g1 (Dz3GT1) and c66977_g1 (Dz3GT2) are identified as sterol 3-*O*-glucosyltransferases in our previous study (Li et al., 2018).

The deduced amino acid sequences of the 32 putative UGT genes and two sterol 3-*O*-glucosyltransferases Dz3GT1 and Dz3GT2 from *D. zingiberensis* were aligned with β-solanine/β-chaconine rhamnosyltransferase StSGT3, soyasaponin III rhamnosyltransferase GmSGT3, and sterol 3-*O*-glucosyltransferases from other plants using the CLUSTAL W. The phylogenetic tree was constructed by the neighbor-joining method of the MEGA 7.0 program (Kumar et al., 2016) and modified by iTOL (Letunic and Bork, 2019). The bootstrap value was 1,000. The information of the UGTs used in the phylogenetic analysis is summarized in Supplementary Table 1. The UGTs clustered with StSGT3 and GmSGT3 were chosen as UGT candidates in this study.

### Isolation of the Full-Length cDNAs of UGT Candidates

Total RNAs from the rhizomes of *D. zingiberensis* were isolated by EASYspin Plus RNA Extraction Kit (Aidlab, China) and treated with DNase I (Thermo Fisher Scientific, United States) to remove genomic DNA contaminations. The first-strand cDNA was synthesized with the RevertAid First Strand cDNA Synthesis Kit (Thermo Fisher Scientific, United States).

The full-length cDNAs of UGT candidates were amplified by standard RT-PCRs, and the PCR products were ligated into the pMD18-T vector (Takara, China) for sequencing confirmation. The primers used are listed in Supplementary Table 2.

### Expression and Purification of Recombinant Proteins in *Escherichia coli*

The full-length *DzGTs* were sub-cloned into the *Bam*HI and *Eco*RI restriction sites of expression vector pGEX-2T, which encodes an N-terminal GST-tagged fusion protein of DzGTs by ClonExpress^®^ II One Step Cloning Kit (Vazyme, China). The recombinant expression vectors were transformed into *E. coli* BL21 competent cells. The transformed *E. coli* cells were grown at 37°C in Luria–Bertani (LB) medium containing 100 μg/mL of ampicillin until OD_600_ reached 0.4–0.6. Next the protein expression was induced with the final concentration of 1 mM isopropyl-1-thio-β-D-galactopyranoside (IPTG) in the medium at 16°C for 20 h on a rotary shaker (220 rpm). To purify the recombinant proteins, cells were collected by centrifugation, suspended in 50 mM Tris-HCl buffer (pH 8.0) and disrupted using a sonicator. Following the manufacturer’s protocol, the recombinant DzGTs were purified using the Glutathione Sepharose 4B kit (GE Healthcare, United States). The primers used for the cloning are listed in Supplementary Table 2. The purified proteins were analyzed by SDS-PAGE, and protein quantification was determined by Bradford assays.

### Enzyme Assays

Enzyme assays of the recombinant UGTs were performed in a 200 μL reaction mixture containing 50 mM Tris-HCl buffer (pH 8.0), 2 mM dithiothreitol (DTT), 0.5 mM sugar donors, 150 μM substrates and 5 μg of the purified UGTs. Control reactions were performed by omitting the purified UGTs. After incubating the reaction mixture overnight at 30°C, the mixture was extracted three times with 600 μL ethyl acetate and evaporated to dryness at room temperature. The samples were redissolved in 400 μL methanol and subjected to HPLC and LC-MS analysis. The experiments were performed in three independent repeats.

To analyze the kinetic parameters (*K*_m_ and *V*_max_) of recombinant DzGT1, a 200 μL reaction mixture containing 50 mM Tris-HCl buffer (pH 8.0), 0–320 μM trillin, 2 mM DTT, 1 mM UDP-rhamnose, and 1 μg of the purified DzGT1 was prepared. The reaction mixture was prewarmed at 30°C, and the reaction was initiated by adding the prewarmed DzGT1. After incubating the reaction mixture for 1 h at 30°C, it was stopped with 200 μL of methanol prior to HPLC analysis. The experiments were performed in three independent repeats. The enzyme velocity was calculated based on the content of the product PSA produced at various concentrations of substrate through HPLC detection. Next, the kinetic parameters were determined by nonlinear regression analysis using the GraphPad Prism 5.01 software by creating an XY data table first and entering substrate concentration into X, and enzyme velocity into Y. After entering data, the Analyze option, nonlinear regression, the panel of enzyme kinetics equation, and Michaelis–Menten were chosen in sequence to fit a substrate–velocity curve to determine the *V*max and *K*m.

### HPLC and LC-MS Analysis

High-performance liquid chromatography analysis was performed on an LC-20AT instrument (Shimadzu, Japan) with an inertsil ODS-SP reverse phase column (250 mm *×* 4.6 mm, 5 μm) at 30°C. The wave-length was set to 203 nm. The Milli-Q water (solvent A) and HPLC-grade acetonitrile (solvent B) were used as the mobile phase, and samples were separated as follows with a flow rate of 0.8 mL/min: 0–20 min, 10–90% B; 20–30 min, 90% B; 30–31 min, 90–10% B; 31–40 min, 10% B. For LC-MS analysis, samples were detected by LC-30AD with a triple quadrupole mass spectrometer LCMS-8060 (Shimadzu, Japan) and an electrospray ionization source. ACQUITY UPLC BEH C18 column (100 mm × 2.1 mm, 1.7 μm) was used, and the oven temperature was set at 40°C. The Milli-Q water (solvent A) and HPLC-grade acetonitrile (solvent B) were used as the mobile phase, and samples were separated as follows: 0–15 min, 20–60% B; 15–17 min, 60% B; 17–25 min, 60–90% B; 25–30 min, 90% B; 30–35 min, 90–20% B; 35–40 min, 20% B and the flow rate was 0.3 mL/min. MS data were acquired in a positive ion mode with the ranges of m/z 100–800 and the collision energy was set to 25 eV.

### Bioinformatics Analysis of DzGT1

The molecular weight and theoretical isoelectric point were determined using expasy tools.^1^ Conserved domain search tools were used to find conserved motifs and domains of proteins.^2^ The signal peptide was predicted using SignalP-5.0.^3^ The transmembrane topology was analyzed using TMHMM2^4^ and DeepTMHMM.^5^ ProteinBLAST^6^ was performed to obtain the homologs of DzGT1. The amino acid sequences of DzGT1 and some known rhamnosyltransferases were aligned and analyzed using Clustal Omega.^7^

### Gene Expression and Metabolite Analysis in *D. zingiberensis*

The healthy rhizomes were planted at 25°C with 14/10 h of a light/dark cycle. After three months, *D. zingiberensis* materials (rhizomes, leaves, and stems) were collected to measure the transcript abundance of *DzGT1* and PSA content.

To detect the PSA concentration in different organs, plant materials from rhizomes, leaves, and stems were ground into fine powder in liquid nitrogen and dried at 37°C. About 20 mg of dried plant materials were extracted thrice with 1 mL of methanol under sonication (180 W, 40 kHz, 30°C, 20 min). The methanol extracts were air-dried at room temperature and redissolved in 1 mL methanol for HPLC analysis. Three biological repeats were performed. To determine calibration curve, different concentrations (1–80 μg/mL) of PSA were used. Calibration curve was constructed by plotting the peak area versus the concentration of PSA, using linear regression analysis. The concentrations of PSA in rhizomes, leaves, and stems were quantified by relating the respective peak area to the regression line.

To monitor the gene expression, total RNA from the plant material powders under liquid nitrogen were isolated by plant RNA Extraction kit (Aidlab, China) and treated with DNase I (Thermo Fisher Scientific, United States) to remove genomic DNA contaminations. The first-strand cDNA was synthesized with the SuperScript III reverse transcriptase (Invitrogen, United States). FastStart Universal SYBR Green Master mix (Rox) (Roche, Germany) was used to do the quantitative RT-PCR (qRT-PCR) with an ABI 7500 Fast Real-Time PCR Detection System. The PCR was performed as follows: 10 min of initial denaturation at 95°C, followed by 40 cycles of 95°C for 15 s and then 55°C for 1 min. All real-time PCRs were performed in three biological repeats. The primers used are listed in Supplementary Table 2.

The correlation between PSA content and the relative expression of *DzGT1* was analyzed by Pearson correlation in JMP Pro 16 (SAS Inc., United States). The preliminary prediction of trend was analyzed by Fit Y by X platform of JMP Pro 16. The confidence interval was set as 99%.

## Results

### Isolation and Sequence Analysis of Putative UGT cDNA Candidates

**FIGURE 2:**
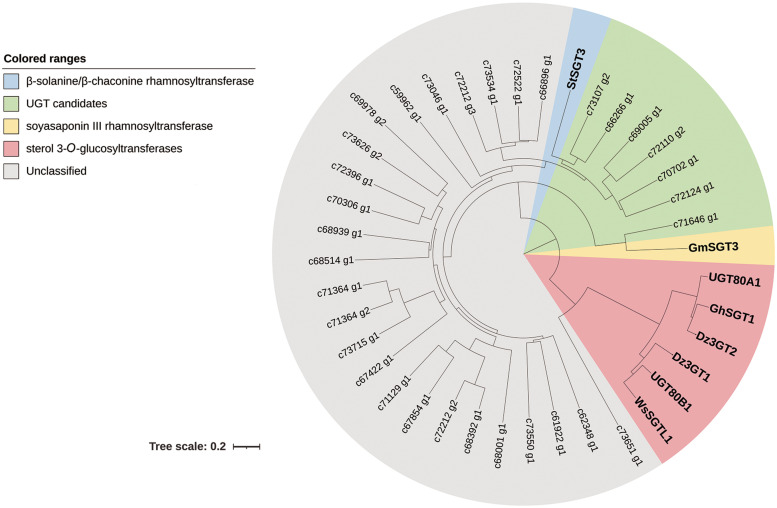
Phylogenetic analysis of the UGT candidates from *Dioscorea zingiberensis* with other related function known sequences. The tree was constructed by the neighbor-joining method of MEGA 7.0, modified by iTOL, and the bootstrap values was 1,000. The scale bar represents 0.2 amino acid substitutions per site.

A phylogenetic tree was constructed to examine the relationships of the 32 putative UGTs and two sterol 3-*O*-glucosyltransferases from *D. zingiberensis* with rhamnosyltransferases StSGT3 and GmSGT3 as well as sterol 3-*O*-glucosyltransferases from other plants (Figure 2). Out of 32 putative UGTs, c73651_g1 occurred in the same branch with sterol 3-*O*-glucosyltransferases, suggesting that it might be a sterol 3-*O*-glucosyltransferase. c66266_g1, c69005_g1, c70702_g1, c72110_g2, c72124_g1, and c73107_g2 clustered with β-solanine/β-chaconine rhamnosyltransferase StSGT3 and c71646_g1 showed a closer relationship to soyasaponin III rhamnosyltransferase GmSGT3, implying possible roles for c66266_g1, c69005_g1, c70702_g1, c72110_g2, c72124_g1, c73107_g2, and c71646_g1 as UDP-rhamnosyltransferases involved in the biosynthesis of dioscin.

The full-length cDNAs of these seven UGTs were amplified by RT-PCRs, and *c69005_g1*, *c66266_g1*, *c70702_g1*, and *c73107_g2* (designated as *DzGT1*-*DzGT4*) were successfully cloned. The sequences were confirmed by Sanger sequencing, and their GenBank accession numbers are MW971922-MW971925. The deduced amino acid sequences of the four UGT candidates showed 38.7%-51.6% identity with each other, and the amino acid sequence alignment results were shown in Supplementary Figure 1. All of them have the 44-amino acid consensus sequence near the C-terminal, referred to as the plant secondary product glycosyltransferase (PSPG) box (Paquette et al., 2003; Rahimi et al., 2019; Zhang et al., 2020), which is highlighted by red color (Supplementary Figure 1).

### *In vitro* Enzyme Assays

To examine the enzyme activities of recombinant UGTs, DzGT1-DzGT4 were successfully expressed in *E. coli* BL21 cells and purified using the Glutathione Sepharose 4B kit (GE Healthcare, United States). The molecular weight of the recombinant DzGT1-DzGT4 was approximately 80 kDa, which was consistent with their predicted molecular weight (Supplementary Figure 2).

**FIGURE 3:**
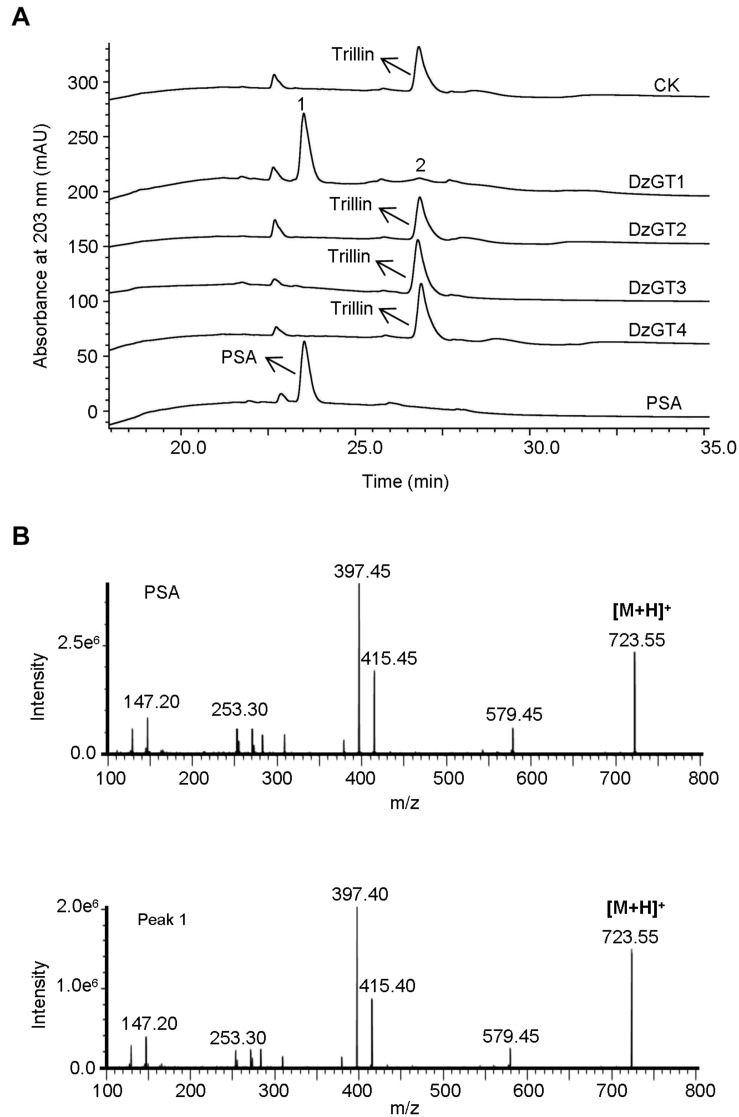
HPLC and LC-MS analysis of the enzyme activities of the purified recombinant DzGTs to trillin i*n vitro*. **(A)** The enzyme activity of the recombinant DzGT1 was shown for the conversion of most trillin to prosapogenin A of dioscin (PSA) (peak 1) and a little trillin (peak 2) unconverted. Compared to the control, no new product peak was detected when DzGT2-DzGT4 were assayed. **(B)** The mass spectra of the peak 1 with its corresponding authentic standard PSA.

The purified recombinant DzGTs were first assayed *in vitro* using the substrate trillin and the sugar donor UDP-rhamnose. Out of the four DzGTs, only DzGT1 showed the activity to trillin. The recombinant DzGT1 could glycosylate most of the substrate trillin to form a new product peak (peak 1), and only a little trillin (peak 2) was not converted (Figure 3A). The LC-MS detection result showed that the molecular weight of the new product is 723.55 [M + H]^+^, which is consistent with the molecular weight of PSA (Figure 3B). The retention time (Figure 3A) and mass spectra of peak 1 were consistent with chemical standard PSA (Figure 3B), showing DzGT1 is a trillin rhamnosyltransferase. Other DzGTs had no activity to trillin when UDP-rhamnose was used (Figure 3A).

The substrate specificity of DzGT1 was determined using UDP-rhamnose as the sugar donor, and PSA, dioscin, gracillin, deltonin, and zingiberensis newsaponin were the acceptors. The results showed that none of them was converted by the recombinant DzGT1. In addition, other glycosyl donors UDP-glucose, UDP-galactose, or UDP-glucuronic acid, were used to detect the activities of DzGT1 to substrates of trillin, PSA, dioscin, gracillin, deltonin, and zingiberensis newsaponin. The results showed DzGT1 had no activity to those substrates using these sugar donors (Table 1). The results showed none of them was converted by the recombinant DzGT1. The enzyme activities of DzGT2-DzGT4 to the substrate PSA were detected using UDP-rhamnose as the sugar donor. The results showed DzGT2-DzGT4 had no activity to PSA (data not shown). The structures of the substrates used for the enzyme assays were shown in Supplementary Figure 3.

**TABLE 1:** Enzymatic activities of the purified recombinant DzGT1 toward a range of substrates *in vitro*.

Substrate	Enzyme activity			
	
	UDP-rhamnose	UDP-glucose	UDP-galactose	UDP-glucuronic acid
Trillin	Yes^a^	ND	ND	ND
Prosapogenin A of dioscin	ND^b^	ND	ND	ND
Dioscin	ND	ND	ND	ND
Gracillin	ND	ND	ND	ND
Deltonin	ND	ND	ND	ND
Zingiberensis saponin	ND	ND	ND	ND

*^*a*^Yes shows when UDP-rhamnose was used as a sugar donor, DzGT1 had the enzyme activity to substrate trillin. ^*b*^ND means“not detected.”*

Besides, the kinetic parameters for DzGT1 with different concentrations of trillin were measured using 1 mM UDP-rhamnose as the sugar donor. The kinetic parameters were determined by nonlinear regression analysis using the GraphPad Prism 5.01 software. According to the analysis, the *K*_m_ and *V*_max_ were 13.05 ± 2.17 μM and 0.24 ± 0.01 μmol/L/min, respectively. The substrate–velocity curve was shown in Supplementary Figure 4.

### Predicted Properties of DzGT1

The putative conserved domain search results showed that the DzGT1 protein has specific hits with GTs and belongs to the Glycosyltransferase GTB-type superfamily. The active sites (Gly-23, His-24, Ile-26, Phe-188, Glu-191, Leu-192, Leu-196, Asp-234, Ser-295, His-366, Gly-368, Asn-370, Ser-371, and Glu-374), acceptor substrate pocket (His-24), TDP-binding sites (Gly-23, Ser-295, His-366, Gly-368, Asn-370, Ser-371) were identified (Supplementary Figure 5A). The molecular weight of the DzGT1 protein is 53.1 kDa with an isoelectric point of 5.45. The DzGT1 was found to have neither signal peptide by SignalP-5.0 (Supplementary Figure 5B) nor transmembrane topology by TMHMM2 and DeepTMHMM (Supplementary Figures 5C,D).

### Alignment Results

BlastP analysis showed that the closest homolog of DzGT1 in the NR database is scopoletin glucosyltransferase-like (78.0%) from *Dioscorea cayenensis* subsp. *rotundata*, whose function has not been characterized. The enzyme with the highest identity to known proteins is flavonol-7-*O*-glucosyltransferase UGT703B1 (46.8%) from *Crocus sativus* (Ahrazem et al., 2014). DzGT1 was also aligned with some UDP-rhamnosyltransferases from other plants. DzGT1 has 35.1% amino acids identity to β-solanine/β-chaconine (steroidal glycoalkaloid) rhamnosyltransferase StSGT3 from potato (McCue et al., 2007), 28.2% amino acids identity to soyasaponin III (triterpene saponin) rhamnosyltransferase GmSGT3 from soybean (Shibuya et al., 2010) and 27.2% amino acids identity to 17-hydroxygeranyllinalool (diterpene) rhamnosyltransferase UGT91T1 from *Nicotiana attenuata* (Heiling et al., 2021). In addition, DzGT1 shows 22.4-36.2% amino acid identity to these flavonoid UDP-rhamnosyltransferases UGT78D1 (Jones et al., 2003), UGT76F1 (Liu et al., 2018), UGT79B2 (Li et al., 2017), UGT91L1 (Casas et al., 2016), UGT89C1 (Zong et al., 2019), and EkF3URhaT (Lyu et al., 2020). These results demonstrated that DzGT1 displays low amino acid sequence identities to other known rhamnosyltransferases.

### The Relevance of *DzGT1* Transcripts and PSA Accumulation in *D. zingiberensis*

**FIGURE 4:**
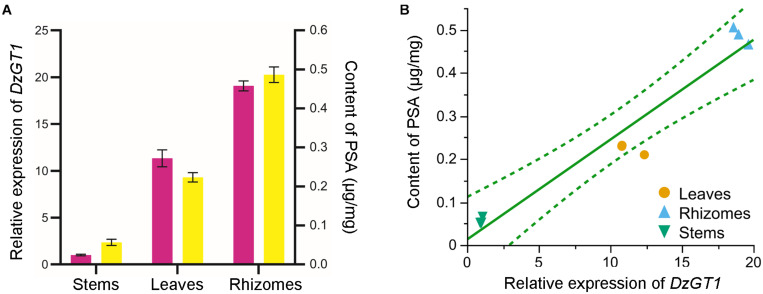
Correlation analysis of the *DzGT1* transcript abundance with the accumulation of the prosapogenin A of dioscin (PSA) in *Dioscorea zingiberensis*. **(A)** The relative transcript level of the *DzGT1* (magenta bar) and the content of PSA (yellow bar) in the different organs of *D. zingiberensis*. The *D. zingiberensis* actin gene was used as an internal standard for the gene expression analysis. Values are means ± SD of three biological replicates. Data was analyzed by GraphPad Prism 5.01 software. **(B)** The correlation between the content of PSA and the relative expression of *DzGT1* analyzed by Fit Y by X platform of JMP Pro 16. The linear fit equation is Y = 0.012629 + 0.0231855 *X and *R*^2^ = 0.940799, showing the high correlation between PSA content and the relative expression of *DzGT1*. The dotted lines display a 99% confidence interval.

The calibration curve of PSA showed good linearity (*R*^2^ = 0.9986) (Supplementary Figure 6). PSA was mostly accumulated in the rhizomes (0.49 ± 0.02 μg/mg dry weight), and the content of PSA was lower in leaves (0.22 ± 0.01 μg/mg dry weight), while the lowest concentration of PSA was detected in stems (0.06 ± 0.01 μg/mg dry weight) (Figure 4A). Consistent with the metabolite accumulation, the *DzGT1* transcript was highest in the rhizomes, whereas the relative low expressions were found in the stems and leaves (Figure 4A). We first tested whether the data fit the normal distribution, and the result of goodness of fit test was not significant, so we accepted the hypothesis of normal distribution of the data. Then we did Pearson correlation test whose coefficient was 0.97, showing high correlation between the content of PSA and the relative expression of *DzGT1*. Finally, we gave a preliminary prediction of the trend by linear regression with Fit Y by X platform of JMP Pro software. The linear fit equation is Y = 0.012629 + 0.0231855X and *R*^2^ = 0.940799 (99% confidence interval), and the value of *R*^2^ showed that the model had a high fitting degree to the data (Figure 4B). Thus, the *DzGT1* transcripts match the accumulation pattern of the metabolite PSA in *D. zingiberensis* in different organs, supporting the proposed biochemical role of DzGT1 in PSA biosynthesis *in vivo*.

## Discussion

There are many reports about the pharmacological activities of dioscin, but genes encoding UDP-rhamnosyltransferase involved in dioscin biosynthesis have not been studied yet. The phytochemical study has elucidated the presence of dioscin in *D. zingiberensis*, which allows it to be a suitable material for the molecular investigation of glycosylation. In this study, we isolated four genes and confirmed a UDP-rhamnosyltransferase gene *DzGT1* from *D. zingiberensis*. Heterologous expression in *E. coli* showed that DzGT1 exhibited an enzyme activity that glycosylated the substrate trillin to form the product PSA.

Based on these results, we speculate that dioscin is most likely synthesized by the first route (Figure 1). Firstly, diosgenin is glycosylated by sterol 3-*O-*glucosyltransferase to form the product trillin, which is converted into PSA by DzGT1. Secondly, dioscin is synthesized by another unidentified rhamnosyltransferase adding a rhamnose onto PSA. But the possibility of dioscin being biosynthesized by the second route cannot be ruled out. The rhamnosyltransferase, which could convert trillin to PSB may also exist, but the gene has not been isolated. The function of DzGT1 needs to be studied further in *D. zingiberensis* by gene knockout experiment to identify its role *in vivo* in the future.

Moreover, the identification of DzGT1 provides the gene resource for PSA production in microbes. It also gives the basis for heterologous biosynthesis of dioscin and relevant steroidal saponins in other plants and microbial hosts. Diosgenin was successfully synthesized in the cholesterol-producing yeasts (Christ et al., 2019), and sterol 3-*O*-glucosyltransferases, converting diosgenin to trillin, have been isolated (Ye et al., 2017; Li et al., 2018). Integrating the sterol 3-*O*-glucosyltransferase gene and *DzGT1* into the diosgenin-producing yeasts, PSA is most likely synthesized. Pharmacological studies show that PSA has antiplatelet aggregation and anti-cancer activities (Wang et al., 2014; Mbaveng et al., 2020), and it can be developed as a new drug in the future. However, the biosynthetic pathway of the sugar chain of dioscin has not been elucidated completely. Future work on gene mining is needed to provide the gene resource for the heterologous biosynthesis of dioscin.

BlastP analysis shows that the closest homolog of DzGT1 to known proteins is flavonol-7-*O*-glucosyltransferase UGT703B1 (46.8%) from *C. sativus* (Ahrazem et al., 2014). DzGT1 was also aligned with some functional UDP-rhamnosyltransferases from other plants, showing only 22.4–36.2% amino acid identity to those UDP-rhamnosyltransferases. Although the chemical structures of steroidal saponins are similar to steroidal glycoalkaloids and triterpenes, the amino acid identity of DzGT1 to StSGT3 or GmSGT3 is not high. DzGT1 has 35.1% amino acids identity to StSGT3 (steroidal glycoalkaloid rhamnosyltransferase) from potato (McCue et al., 2007) and only 28.2% amino acids identity to GmSGT3 (triterpene saponin rhamnosyltransferase) from soybean (Shibuya et al., 2010). Furthermore, DzGT1 displays low amino acid sequence identities to other flavonoid UDP-rhamnosyltransferases too. These results show that DzGT1 exhibits low amino acid sequence identities to other known rhamnosyltransferases, even lower than glucosyltransferases. Thus, it is difficult to distinguish the rhamnosyltransferases from other glycosyltransferases by sequence analysis.

In addition, the crystal structures of plant rhamnosyltransferase UGT89C1 and its complex with sugar donor UDP-rhamnose were determined (Zong et al., 2019). Based on the structure and site-directed mutagenesis, the results showed Pro-147, Ile-148, Trp-335, Asp-356, and His-357 involved in sugar donor recognition and the detailed interactions with the UDP-rhamnose (Zong et al., 2019). We speculate that the corresponding residues Ala-148, Ile-149, Trp-369, Glu-390, and Gln-391 of DzGT1 might be necessary for recognition and binding of UDP-rhamnose. Besides, the putative conserved domain search results showed that the DzGT1 protein belongs to the Glycosyltransferase GTB-type superfamily and the active sites (Gly-23, His-24, Ile-26, Phe-188, Glu-191, Leu-192, Leu-196, Asp-234, Ser-295, His-366, Gly-368, Asn-370, Ser-371, and Glu-374), acceptor substrate pocket (His-24), UDP-binding sites (Gly-23, Ser-295, His-366, Gly-368, Asn-370, and Ser-371) were identified. Some site-directed mutation experiments could be done to determine the key amino acids on enzyme activity as well as the recognition and binding of sugar donors in the future.

The relevance of *DzGT1* transcripts and PSA accumulation in *D. zingiberensis* was studied too. PSA primarily accumulated in the rhizomes, which is consistent with the *DzGT1* transcripts in different organs (Figure 4A) and the correlation analysis between the PSA content and the relative expression of *DzGT1* displayed their high relevance (Figure 4B). The results of enzyme biochemical property *in vitro*, gene expression and metabolite accumulation *in vivo* supported the proposed role of DzGT1 in PSA biosynthesis in *D. zingiberensis*. However, to clarify the function of *DzGT1 in vivo*, the gene knockout experiment in *D. zingiberensis* is needed in the future.

In conclusion, our study demonstrated that DzGT1 is a UDP-rhamnosyltransferase that could catalyze the substrate trillin to form the product PSA by supplying the sugar donor UDP-rhamnose. The identification and characterization of DzGT1 and the potential metabolic pathway for dioscin biosynthesis in this study provide the basis for heterologous pathway engineering and efficient production of PSA, dioscin, and relevant steroidal saponins in other plants and microbial hosts.

## Data Availability Statement

The datasets presented in this study can be found in online repositories. The names of the repository/repositories and accession number(s) can be found in the article/Supplementary Material.

## Author Contributions

YZ and JL designed the study with the input from SY for bioinformatics analysis. JL performed the gene cloning, biochemical reactions, HPLC, and LC-MS analysis and wrote the manuscript. IM collected the plant materials, performed real-time PCR, and assisted in HPLC analysis. HL and YW performed the bioinformatics analysis. CL assisted in HPLC analysis. SY and IM revised the manuscript. All authors contributed to the article and approved the submitted version.

## Conflict of Interest

The authors declare that the research was conducted in the absence of any commercial or financial relationships that could be construed as a potential conflict of interest.

## Publisher’s Note

All claims expressed in this article are solely those of the authors and do not necessarily represent those of their affiliated organizations, or those of the publisher, the editors and the reviewers. Any product that may be evaluated in this article, or claim that may be made by its manufacturer, is not guaranteed or endorsed by the publisher.

## Footnotes

1
https://web.expasy.org/compute_pi/

2
https://www.ncbi.nlm.nih.gov/Structure/cdd/wrpsb.cgi

3
http://www.cbs.dtu.dk/services/SignalP/

4
https://services.healthtech.dtu.dk/service.php?TMHMM-2.0

5
https://dtu.biolib.com/app/DeepTMHMM/

6
https://blast.ncbi.nlm.nih.gov/Blast.cgi?PROGRAM=blastp&PAGE_TYPE=BlastSearch&LINK_LOC=blasthome

7
https://www.ebi.ac.uk/Tools/msa/clustalo/

## Supplementary Material

Supplementary Figure 1 The amino acid sequence alignment of DzGT1-DzGT4.

Supplementary Figure 2 SDS-PAGE electrophoresis analysis of the purified recombinant DzGTs.

Supplementary Figure 3 Structure of the substrates used for the enzyme assays of DzGT1.

Supplementary Figure 4 Nonlinear regression analysis of DzGT1 with trillin using the GraphPad Prism 5.01 software.

Supplementary Figure 5 Bioinformatic analysis of DzGT1.

Supplementary Figure 6 The calibration curve of PSA.

Supplementary Table 1 Information of the glycosyltransferases used in the phylogenetic analysis.

Supplementary Table 2 Primers used for the gene amplifications in this study.
