# Supplementary material for: Identification and Characterization of a Trillin Rhamnosyltransferase From Dioscorea zingiberensis
Source: Front Plant Sci. 2021 Aug 6;12:713036. doi: 10.3389/fpls.2021.713036 (PMC8377597; doi:10.3389/fpls.2021.713036)
Supplement: Supplementary Figure 1 — The amino acid sequence alignment of DzGT1-DzGT4. [file Data_Sheet_1.pdf]

Supplementary Material

1 Supplementary Figures and Tables

1.1 Supplementary Figures

```
DzGT1      MATHAGHEPRPLRVFFIPFFATGHMIPMVDIARLFAARGVDATVLVTPSNAALISRTIDD 60
DzGT3      -MGTETKTQVPLRVFFFPLMSPGHMIPMIDVAKLFARHGAATTIITTPANEPFARPAIDR 59
DzGT2      ---MTSKAKDNLHILFFPFMAGGHIIPTLDLAKAFATHGVTTTILTTPSYSPLIQRTINH 57
DzGT4      -MGSNSTTPHNLHILFFPFMAPGHAIPMLDMTKLFALHGVRTTFITTPANAPLVQPTINR 59
                      *:::*:*::: ** ** :*::: ** :*. :*.:.**:   :   :*:

DzGT1      AAA----SGLPIRTLLYPFPSAEV-GLPPGAENIASVPLADAPKVD-AASLLTAGDHDRL 114
DzGT3      ANSSSPPLPHPINLIFLPFPSSVHHLLPAGHENFSSITSDDDFNKLFTALFALDTPFGHL 119
DzGT2      YS-----GQLPIHLSLISFPTTIS-GLPSGCDSVTSLNSQHERIIFLCSLSSLCQPFKRA 111
DzGT4      ANSANP-TAPPIKLTLIPFPSAEV-GLPSGCENAQSLESLDMRSNFYKAVMMLRQPFDQV 117
                     **.  :  **::    ** * :.  *:   .       :       . :

DzGT1      LRLHHPDAVVSDTHFAWTTSIARDLRIPRITFHAIGLFPVCVLGSLIRNLPHLSVTDD-V 173
DzGT3      LQSHQPDCLVSDSVFTWSAAISADLNIPRLIFHGPGIFTLCIHDNLNLHDV-----YGQA 174
DzGT2      LTELRPDCIITDMFFPWTSAVARELQIPRLVFQSESFFSLCMTDSLLRYKPHLTISDDID 171
DzGT4      LKELHPDAIVTDSFLPWTTDAASEFGIPRLVFHGTSSFALCAWHNVDEYNPHE----RPE 173
           *   :**.:::*  : *::  : :: ***: *:. . * :*   .:

DzGT1      HPFIVPDLPHPVQMFRPELPDFLRSENTAIAQAMSALNEAEAGSLGVVVNSFAEIESAYA 233
DzGT3      TTFTIQDIPHDIHMNRWEVPNIFTL-P----MWLQMLRDAEDKSYGVVVNSFYELEPDYA 229
DzGT2      ESFILPGLPHPIKMSKSQILDPTKI-TPEFSELFSLMGESAAESYGVVVNSFLELEPEYA 230
DzGT4      ESFTIPGLPHRIHMLKSQVPIIPKT-NPSFTDVMRAVKESDAKSYGAVVNSFYELEPEYV 232
             * : .:** ::* : ::              :  : ::   * *.***** *:*  *.

DzGT1      DCYYKFDNMRTWFVGPVALADGGEKASRGAAADAKAVANRELCMRWLDKQRERSVVYACF 293
DzGT3      ELYRRKPGRRAWYIGPVSLYNESADEK--AVR-GLAETEHEELTSWLDTKPAGSVVYVSF 286
DzGT2      KHYREFIGRRAWHVGPVSLCSQRVGDD--DDA---SKDHYTECLKWLDSKKPGSVVYVCF 285
DzGT4      DHYRNVMGRRAWYVGPVSLCNEEVVDL--AGRGGEAVIDQDECLNWLDNKQPNSVLYVCF 290
           . * .  . *:*.:***:* .              :  .      ***.:   **:*..*

DzGT1      GSWCHFSAAQLRELALGLEAAGHPFLWVVRDDGE--EWMPEGFEEKLGEKGLVVRGWAPQ 351
DzGT3      GSLCVFSVDQVEEIAAGLEISGHCFVWVVRDKGPST---PEGFEERVKGRGVVVRGWVPQ 343
DzGT2      GSMFSMPSEQLTELAIGLEACEHPFIWVVIRMKGGDHEE-KKVRVLEGGKGVVINGWAPQ 344
DzGT4      GSVGRFTSAQLHEIALGLESSRQPFIWVVKKLFDDEREWLPEYEKRVEGRGLIIRGWAPQ 350
           **   :   *: *:* *** . : *:***              .     :*:::.**.**

DzGT1      VAVLGHDAVGGFVTHCGWNSVLEGVSSGLPMVTWPLSTEQFINEKLVVEVLGTAVRAWEG 411
DzGT3      VVLLGHVAVGGFVNHCGWNSVLEGISAGLPMVTWPLMHEQFINEKLVNEVLGIGVRARRE 403
DzGT2      LVILNHPAVGAFITHCGWNSTMESICAGVPMITWPLFGDQFYNERFIVDVLKVGVEVGVK 404
DzGT4      ILILNHEAVGGFLTHCGWNSALEAVCAGVPMVTMPMLAEQFFNERLLVDVLKIGVGIGVK 410
           : :*.* ***.*:.******.:*.:.:*:**:* *:  :** **::: :**  .*

DzGT1      -------FRSTEEGEKEVVKAGDVAAAVEKVMGGGEEGEKRRVTAREFGEKAKAAVKVGG 464
DzGT3      --G---------QDPTVVIGREEVAGAVRRLMDGGDEAEVRRGKAREYARMARTAIEDGG 452
DzGT2      KFVVD-------EEDMELVKGEDVKKAVERMMGDGDEKESMRRRLMELGEMARRAVEEGG 457
DzGT4      EYGTGQKFGMGTENVSALVCGEDIEKAVAMLMNAGEEAEEMRRRARELRESAKMALEKGG 470
                       :    ::   ::  **  :*. *:* *  *    *  . *: *:: **

DzGT1      SSYEGLSRLIGEIRDWPKKINGCDAATA---       492
DzGT3      SSRVDLNRLMDELTQRRGRVGGEEKVEISSN       483
DzGT2      SSYVDLKNLINELCEVCSEHDMNLSKQ----       484
DzGT4      SSSLDICNLIRELTEKQRPVPSLY-------       494
           **  .: .*: *: :
```

Supplementary Figure 1. The amino acid sequence alignment of DzGT1-DzGT4. The putative plant secondary product glycosyltransferase (PSPG) box is highlighted by red colour.

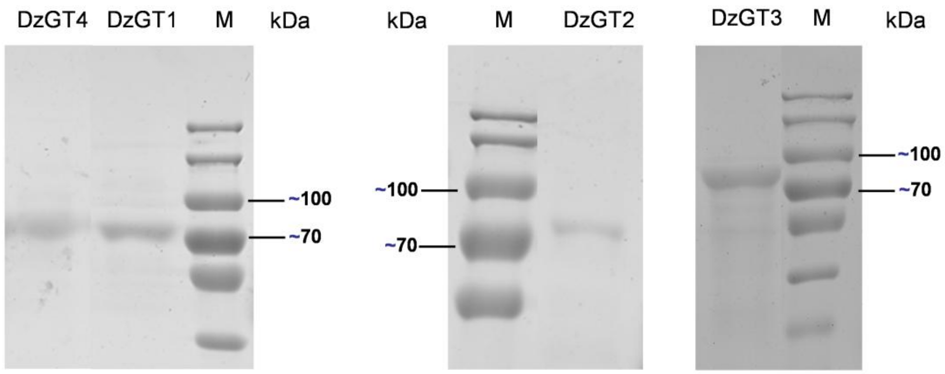

Supplementary Figure 2. SDS-PAGE electrophoresis analysis of the purified recombinant DzGTs.

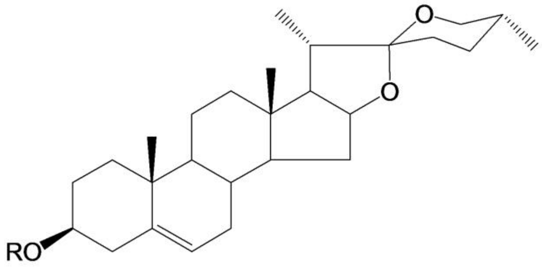

	R
Trillin：	Glc
PSA：	Rha(1→2)-Glc
Dioscin：	Rha(1→4)-[Rha(1→2)]-Glc
Deltonin：	Glc(1→4)-[Rha(1→2)]-Glc
Gracillin：	Glc(1→3)-[Rha(1→2)]-Glc
Zingiberensis newsaponin：	Glc(1→3)-Glc(1→4)-[Rha(1→2)]-Glc

Supplementary Figure 3. Structure of the substrates used for the enzyme assays of DzGT1.

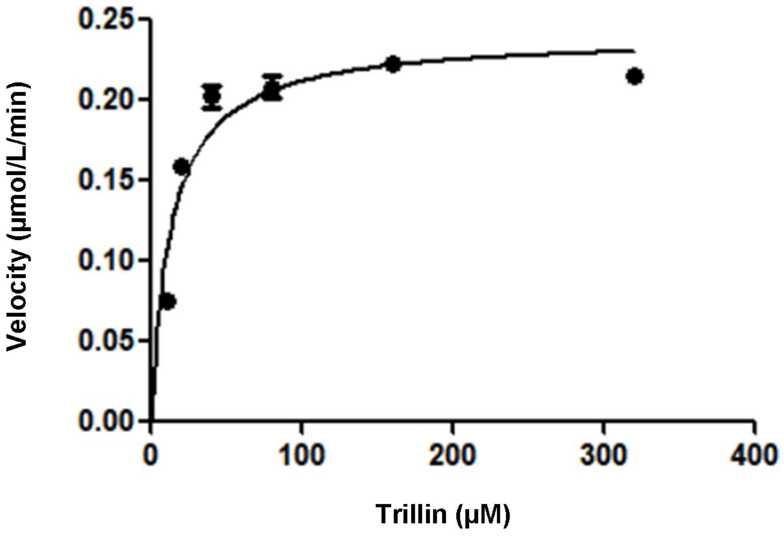

Supplementary Figure 4. Nonlinear regression analysis of DzGT1 with trillin using the GraphPad Prism 5.01 software.The best-fit regression equations is V = 0.24[S]/(13.05 + [S]). V, enzyme velocity (µmol/L/min); [S], substrate (trillin) concentration.

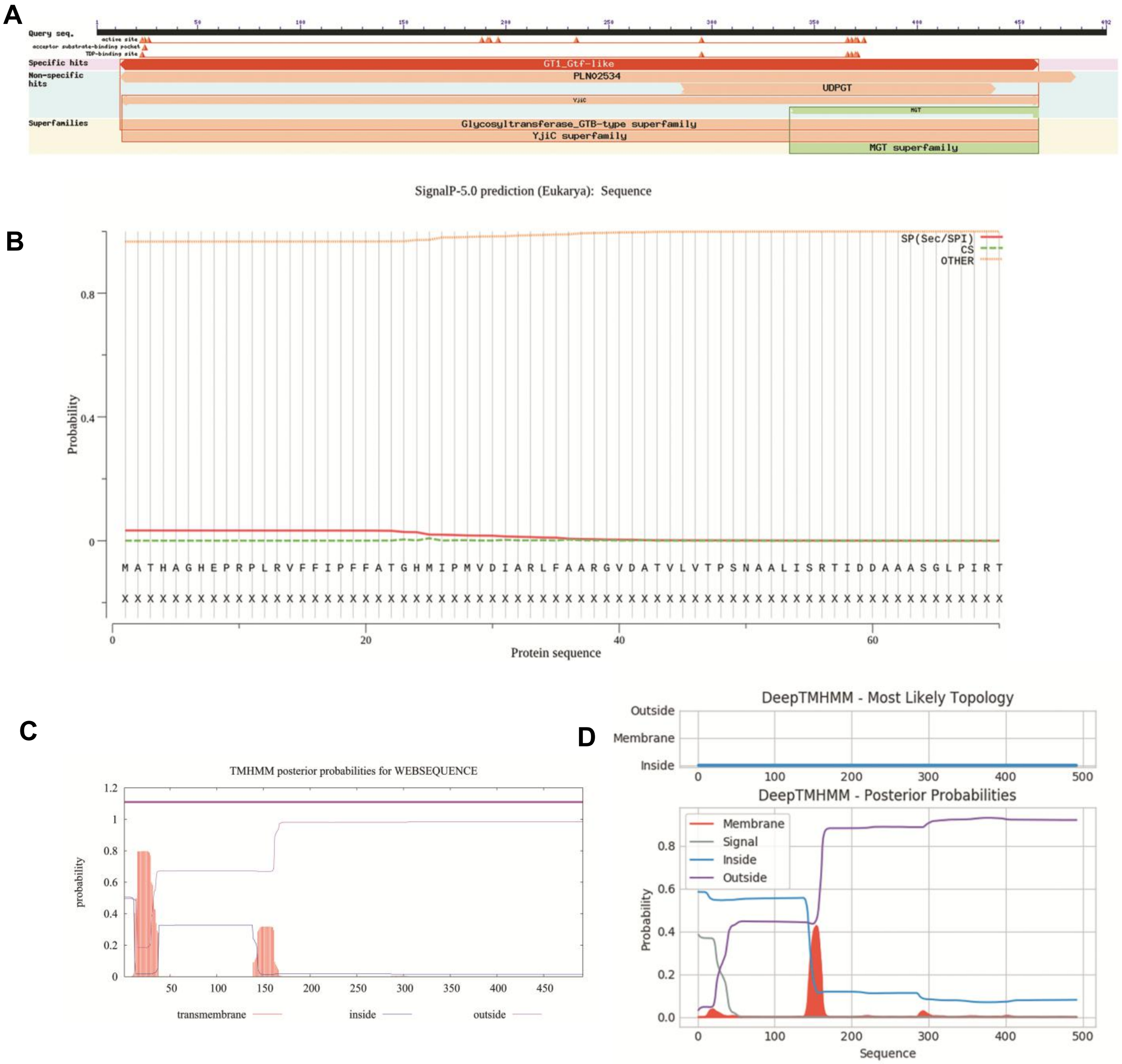

Supplementary Figure 5. Bioinformatic analysis of DzGT1. **(A)** Putative conserved domains of DzGT1 detected by the NCBI conserved domains database. **(B)** Signal peptide prediction of DzGT1 with SignalP 5.0. **(C, D)** Transmembrane topology prediction of DzGT1 with TMHMM2 and DeepTMHMM.

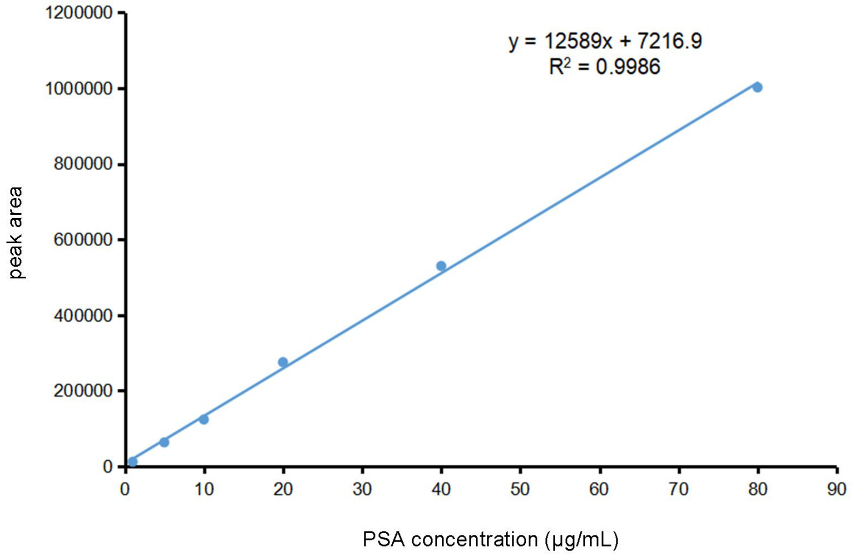

Supplementary Figure 6. The calibration curve of PSA. The linear fit equation is y = 12589x+ 7216.9 and R^2 = 0.9986.

1.2 Supplementary Tables

Supplementary Table 1. Information of the glycosyltransferases used in the phylogenetic analysis

Protein	GenBank accession Number	Plant	Function
UGT80A1	CAB06081	*Avena sativa*	Sterol 3-*O*-glucosyltransferase
UGT80B1	OAP13743	*Arabidopsis thaliana*	Sterol 3-*O*-glucosyltransferase
GhSGT1	AHX00584	*Gossypium hirsutum*	Sterol 3-*O*-glucosyltransferase
SGTL1	ABC96116	*Withania somnifera*	Sterol 3-*O*-glucosyltransferase
Dz3GT1	AVI57699	*Dioscorea zingiberensis*	Sterol 3-*O*-glucosyltransferase
Dz3GT2	AVI57700	*D. zingiberensis*	Sterol 3-*O*-glucosyltransferase
GmSGT3	NP_001240857	*Glycine max*	soyasaponin III rhamnosyltransferase
StSGT3	NP_001274799	*Solanum tuberosum*	β-solanine/β-chaconine rhamnosyltransferase

Supplementary Table 2. Primers used for the gene amplifications in this study

Name	Primers 5' to 3' sequence
For pMD18-T /pGEX-2T	
DzGT1-F	GATCTGGTTCCGCGTGGATCCATGGCCACCCATGCC
DzGT1-R	TCAGTCAGTCACGATGAATTCTCAAGCGGTAGCAGCATC
DzGT2-F	GATCTGGTTCCGCGTGGATCCATGACTTCAAAAGCCAAAGA
DzGT2-R	TCAGTCAGTCACGATGAATTCTTATTGCTTGGACAGATTCA
DzGT3-F	GATCTGGTTCCGCGTGGATCCATGGGGACTGAAACAAAAAC
DzGT3-R	TCAGTCAGTCACGATGAATTCCTAGTTTGAAGAGATTTCTACTTT
DzGT4-F	GATCTGGTTCCGCGTGGATCCATGGGATCCAACAGTACTACT
DzGT4-R	TCAGTCAGTCACGATGAATTCCTAGTATAAACTAGGTACTGGTCT
For real-time PCR	
Actin-F	TGCTGGATTCTGGTGATGGT
Actin-R	CCCGTTCTGCGGTAGTAGTG
DzGT1-F	TTCCTCCGCTCCGAAAACA
DzGT1-R	CGAACCACGTCCGCATATTG
